# Time-Resolved Transcriptomic Profiling of Surgical Wounds Identifies Stage-Specific Therapeutic Targets for Residual Ovarian Cancer

**DOI:** 10.3390/pharmaceutics18040413

**Published:** 2026-03-28

**Authors:** Seongyun Lim, Young-Jae Cho, Myeong-Seon Kim, Jung-Joo Choi, Ji-Yoon Ryu, Jae Ryoung Hwang, Ju-Yeon Choi, Mahesh Chandra Patra, Mohamed El-Agamy Farh, Insuk Sohn, Jeong-Won Lee, Yoo-Young Lee

**Affiliations:** 1 Division of Gynecologic Oncology, Department of Obstetrics and Gynecology, Samsung Medical Center, Sungkyunkwan University School of Medicine, Seoul 06351, Republic of Korea; 2 Research Institute for Future Medicine, Samsung Medical Center, Sungkyunkwan University School of Medicine, Seoul 06531, Republic of Korea; 3 Department of Obstetrics and Gynecology, Chung-Ang University Gwangmyeong Hospital, Chung-Ang University School of Medicine, Seoul 14353, Republic of Korea; 4 Drug Development Team, ARONTIER Inc., Seoul 06735, Republic of Korea

**Keywords:** epithelial ovarian cancer, perioperative wound healing, transcriptome-based drug repurposing

## Abstract

**Background:** The optimal timing of adjuvant chemotherapy after cytoreductive surgery in epithelial ovarian cancer remains uncertain, and perioperative wound-healing responses may transiently create a pro-tumorigenic and drug-resistant microenvironment. This study aimed to characterize time-dependent wound-induced transcriptomic alterations and to identify pharmacologic agents capable of reversing these responses. **Methods:** An ID8 murine ovarian cancer model was used to compare no treatment, anesthesia alone, and anesthesia plus surgical wounding mimicking futile laparotomy. Tumors were collected at baseline, 1 day (T1), 1 week (T2), and 2 weeks (T3) after intervention. RNA sequencing was performed, and wound-specific differentially expressed genes (WsDEGs) were defined by excluding anesthesia- and progression-related signatures. Functional enrichment analyses were conducted, followed by transcriptome-based drug repurposing using the REMEDY platform to identify compounds predicted to reverse wound-induced gene expression profiles. **Results:** Surgical wounding significantly increased tumor burden at T1. Transcriptomic analyses revealed distinct, time-dependent wound-associated programs. At T1, WsDEGs were enriched in inflammatory signaling, coagulation, angiogenesis, and immune cell migration, with Vorinostat and Homoharringtonine identified as top candidates to counteract these signatures. At T2, pathways related to cell survival, adhesion, and morphogenesis predominated, with LY-2090314, Artesunate, and Birinapant emerging as potential modulators. At T3, cell-cycle regulation and lipid metabolic pathways were dominant, and Fulvestrant, Atorvastatin, Imatinib, and ABT-737 were predicted to inhibit these processes. **Conclusions:** Perioperative surgical wounding induces dynamic, stage-specific transcriptomic programs that may promote ovarian cancer progression and alter drug responsiveness. These findings support time-adapted perioperative pharmacologic strategies to optimize postoperative cancer therapy.

## 1. Introduction

Epithelial ovarian cancer (EOC) remains one of the most lethal gynecologic malignancies, with the majority of patients diagnosed at an advanced stage and a 5-year overall survival rate below 50% [1,2,3]. The current standard treatment for advanced EOC includes cytoreductive surgery and cytotoxic chemotherapy with/without target agents [1,2,3].

Following cytoreductive surgery, adjuvant chemotherapy should be initiated as soon as the patient has adequately recovered—ideally within 2 to 4 weeks [4,5]—as recommended by several medical societies that define this window period to optimize outcomes. However, there is ongoing debate regarding the prognostic impact of the interval between surgery and the initiation of adjuvant chemotherapy within this recommended window [6]. Interestingly, one study reported that the effect of this waiting time may differ depending on the extent of residual disease after cytoreductive surgery [7]. For instance, patients with microscopic residual disease appeared less affected by the delay, whereas those with macroscopic residual disease showed significantly worse outcomes with prolonged waiting times. This may be explained by Gompertzian tumor growth models, which suggest that microscopic disease lies in the early phase with low proliferative activity, whereas macroscopic residual disease may fall into the intermediate phase characterized by the steepest growth kinetics—thereby making tumors more vulnerable to treatment delays and potentially leading to worse outcomes [8].

In addition to these intrinsic growth dynamics, the perioperative environment—marked by surgical stress and wound healing responses—can further accelerate the expansion of residual micro- and macroscopic disease. These perioperative biological processes are closely associated with immune regulatory mechanisms within the ovarian cancer tumor microenvironment, which can influence tumor progression and therapeutic responses [9]. This surgery-induced accelerated outgrowth renders such residual tumors especially susceptible to rapid progression, underscoring the importance of timely and targeted postoperative interventions.

This underscores the need to more precisely define the optimal (and potentially shorter) interval for initiating adjuvant chemotherapy following cytoreductive surgery, as accumulating evidence suggests that earlier initiation may be more beneficial, despite the current wide variation in clinical practice. Although theoretical models imply that earlier initiation of adjuvant chemotherapy may enhance clinical outcomes [10], the concept of administering chemotherapy on the same day (the earliest chemotherapy model) as cytoreductive surgery remains unknown. Due to ethical and logistical constraints, clinical trials investigating this approach are limited; thus, insights are primarily derived from preclinical studies. In one animal model, same-day chemotherapy was paradoxically associated with inferior oncologic outcomes compared to administration following the perioperative period [11]. This detrimental effect has been attributed not solely to tumor kinetics, but rather to perioperative biological processes related to wound healing [12,13,14,15], which may promote tumor growth and reduce sensitivity to chemotherapy. Clinical data further support this hypothesis, as procedures such as “futile laparotomy”—surgical exploration without meaningful tumor debulking—have been linked to poorer prognosis, possibly due to pro-tumorigenic effects of surgical wounding alone [14,15,16,17,18,19]. Collectively, these findings suggest that although cytoreductive surgery remains the standard of care, it may concurrently induce wound healing-related biological responses that facilitate residual tumor progression and attenuate the efficacy of subsequent chemotherapy.

This study aims to investigate the transcriptomic changes occurring in tumors during the perioperative period using a wound-healing-associated murine model. By applying in silico analytic approaches to the RNA expression data, the study seeks to identify molecular pathways and gene alterations that may mediate the pro-tumorigenic effects of surgical wounding. Additionally, drug repurposing strategies will be employed to explore candidate compounds with the potential to counteract these deleterious wound-healing-induced responses.

## 2. Materials and Methods

### 2.1. Experimental Design

**Figure 1:**
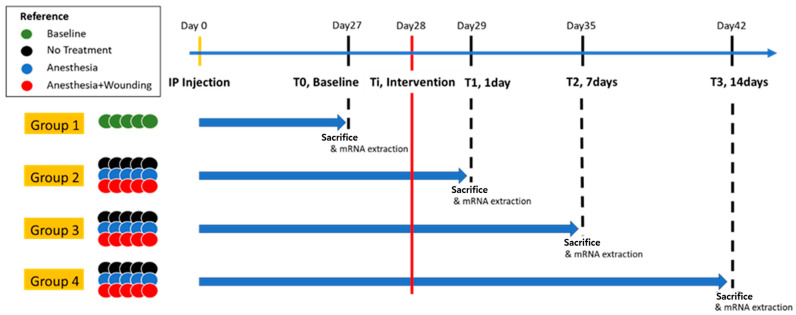
Experimental design and intervention timeline. Tumor-bearing mice were assigned to four experimental groups: baseline control (Group 1), no treatment (Group 2), anesthesia alone (Group 3), and anesthesia plus surgical wounding (Group 4). The timing of intervention (Ti) is indicated by the red line. Tumor growth was monitored longitudinally, and tumors were harvested at predefined time points following intervention to capture temporal changes in tumor growth and transcriptomic profiles.

The overall experimental scheme is illustrated in Figure 1. The study was designed to evaluate the transcriptomic impact of surgical wounding over time. The timeline was defined relative to tumor induction: Day 27, when tumors were sufficiently established, was designated as T0 (Baseline). Day 28, when interventions were performed, was designated as Ti (Intervention). Interventions were classified into three groups: (1) No-treatment (N), (2) Anesthesia only (A), and (3) Anesthesia plus Wounding (AW). The “Wounding” procedure consisted of a 4-cm midline skin incision followed by suturing without tumor removal, designed to mimic a clinical “futile laparotomy” scenario where no meaningful cytoreduction is achieved. Subsequent timepoints were defined as follows: T1 (Day 29, 1-day post-intervention), T2 (Day 35, 1-week post-intervention), and T3 (Day 42, 2-weeks post-intervention). Mice were allocated into four groups for sacrifice and sample collection:

- Group 1 (Baseline): Sacrificed on Day 27 (T0) to serve as a reference control.
- Groups 2, 3, and 4: Sacrificed at T1, T2, and T3, respectively. Within these groups, mice were further subdivided based on the intervention (N, A, AW).

This stratification resulted in a total of 10 subgroups (Base, T1_N, T1_A, T1_AW, T2_N, T2_A, T2_AW, T3_N, T3_A, T3_AW), allowing for the isolation of wound-specific effects by excluding variables related to anesthesia and simple tumor progression.

### 2.2. Cell Line and Animal Care

The mouse ovarian cancer cell line, ID8, was kindly provided by Dr. Anil K. Sood (Department of Cancer Biology, University of Texas M.D. Anderson Cancer Center, TX, USA). Cells were maintained in Dulbecco’s Modified Eagle’s Medium (DMEM; Gibco, Grand Island, NY, USA) supplemented with 4% fetal bovine serum (Welgene, Gyeongsan, Republic of Korea), 0.2% Insulin-Transferrin-Selenium (ITS) Supplement (BD), and 0.1% gentamicin (Sigma, St. Louis, MO, USA) at 37 °C in a 5% CO_2_ atmosphere.

Female C57BL/6 mice (6–8 weeks old) were purchased from ORIENT BIO (Seongnam, Republic of Korea). All animal experiments were approved by the Institutional Animal Care and Use Committee (IACUC) of Samsung Biomedical Research Institute (SBRI) (approved on 15 June 2020, Protocol No. H-A9-003) and conducted in an Association for Assessment and Accreditation of Laboratory Animal Care (AAALAC) International-accredited facility. Ovarian cancer was induced in all mice by intraperitoneal (IP) injection of ID8 cells (1.0 × 10^6^ cells/0.2 mL Hank’s Balanced Salt Solution (HBSS)). Mice were acclimatized for at least one week before the experiments. Mice were randomly assigned to experimental groups. Initially, 10 mice were assigned to each intervention group (N, A, and AW). Mice were sacrificed at predefined time points (T1, T2, and T3) for tumor collection and transcriptomic analysis.

### 2.3. Assessment of Tumor Burden

Tumor burden was assessed by measuring tumor weights at the time of sacrifice, and sample sets were secured for transcriptomic analysis. To evaluate the distribution of tumor weights across different time points and intervention types, scatter plots were generated, and statistical differences between groups were assessed using Student’s *t*-test. Tumor weight measurements were performed in a blinded manner.

### 2.4. RNA Sequencing and Data Preprocessing

Total RNA was extracted and only samples with an RNA Integrity Number (RIN) ≥ 6.0 were included for library preparation and sequencing. Raw reads underwent quality control using FastQC v0.11.9. Low-quality reads and adapter sequences were trimmed using Cutadapt version 4.9, ensuring a Phred score ≥ 30 and a minimum read length of 36 bp. High-quality reads were mapped to the Mus musculus reference genome (GRCm38.p6) using STAR v2.7.6a. Mapping quality, including splice junction and gene body coverage, was assessed using RSeQC v4.0.1. Gene expression levels were quantified using HTSeq v2.0.2. Samples failing predefined quality control criteria were excluded from downstream analyses. All remaining samples included in the analysis passed predefined quality control thresholds, supporting the reliability of the observed transcriptomic patterns despite limited sample sizes in certain groups. Because tumor samples were collected at predefined time points and only samples passing RNA sequencing quality control criteria were included in the final analysis, the number of RNA-seq samples differed across subgroups.

### 2.5. Identification of Differentially Expressed Genes (DEGs)

Raw counts were normalized using the Upper Quartile of Transcript Per Million (TPM-UQ) method. Multiple-testing correction was initially explored using the Storey false discovery rate (q-value) method; however, no genes remained significant after adjustment. Therefore, for this exploratory transcriptomic analysis, differentially expressed genes (DEGs) were defined as those with an absolute fold change ≥ 1.5 (|Log2FC| ≥ 0.58) and a raw *p*-value ≤ 0.05.

To identify wound-specific DEGs (WsDEGs) induced solely by surgical injury, Venn diagram analysis was performed to exclude genes overlapping with the No-treatment (N) and Anesthesia (A) groups. Transcriptomic variations were visualized using Multidimensional Scaling (MDS) and Pearson correlation matrices in R (version 4.2.3; R Foundation for Statistical Computing, Vienna, Austria) using the ggplot2 (version 3.4) and gplots (version 3.1) packages. Volcano plots were generated using Python (version 3.10) scripts. The expression patterns of WsDEGs across timepoints were visualized using z-score based heatmaps. “Top-DEGs” were defined as high-magnitude markers with |Log2FC| ≥ 4 (i.e., FC is 16 folds) and a raw *p*-value ≤ 0.01.

### 2.6. Functional Enrichment Analysis

Functional pathway analysis was performed using the Metascape platform (http://metascape.org, accessed on 18 October 2024). Enriched terms were identified from KEGG Pathway, GO Biological Processes, Reactome, and CORUM databases. Terms with a gene count >3 and *p* < 0.01 were considered significant. q-values were calculated using the Benjamini–Hochberg procedure. Hierarchical clustering was performed based on Kappa scores (similarity > 0.3) to group related terms.

### 2.7. Transcriptome-Based Drug Repurposing (REMEDY)

To identify potential therapeutic agents, we utilized the REMEDY platform based on the LINCS L1000 database (Phase I: GSE92742, Phase II: GSE70138). Query signatures consisting of up- and down-regulated WsDEGs were matched against drug-induced transcriptional signatures using a Kolmogorov–Smirnov (KS)-based connectivity scoring framework. Perturbagens showing significant negative connectivity scores were prioritized, as these compounds are predicted to reverse the wound-induced transcriptional program.

To further refine candidate prioritization, perturbagens were ranked according to their frequency of occurrence within the top 1000 negatively connected signatures across experimental conditions. Statistical enrichment of candidate compounds was evaluated using Fisher’s exact test. Compounds with frequency counts ≤ 3 or without statistical significance (*p* ≥ 0.05) were excluded from further consideration.

**Figure 2:**
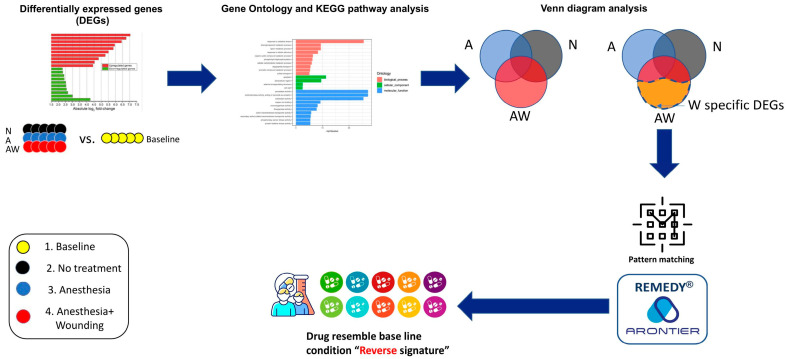
Transcriptomic analysis and drug discovery workflow. Differentially expressed genes (DEGs) were identified by comparing each experimental group (No treatment [N], Anesthesia [A], and Anesthesia plus Wounding [AW]) with baseline tumors. Gene Ontology (GO) and KEGG pathway enrichment analyses were subsequently performed to characterize biological processes associated with perioperative interventions. Venn diagram analysis was used to identify wound-specific DEGs (WsDEGs) by excluding genes shared with the N and A groups. The resulting wound-associated transcriptional signatures were subjected to pattern-matching analysis using the REMEDY^®^ platform based on the LINCS L1000 database to identify candidate compounds predicted to reverse wound-induced gene expression patterns.

Mode of Action (MoA) and clinical phase information for the prioritized compounds were annotated using the Drug Repurposing Hub. The distribution of connectivity scores and ranked perturbagen tables for each time point are provided in Supplementary Figures S1–S3. An overview of the transcriptomic analysis and transcriptome-based drug discovery workflow is shown in Figure 2.

DEGs were identified by comparing each post-intervention group with baseline controls. Gene Ontology and KEGG pathway enrichment analyses were subsequently performed to characterize biological processes associated with anesthesia and surgical wounding. To isolate wounding-specific transcriptional signatures, Venn diagram analyses were used to exclude genes shared with anesthesia-only or no-treatment conditions. These wound-associated gene signatures were then subjected to pattern-matching analysis using the REMEDY^®^ transcriptome-based drug discovery platform to identify candidate compounds predicted to reverse wound-induced gene expression patterns.

## 3. Results

### 3.1. Quality Control and Preprocessing of RNA-Seq Data

A total of 32 samples were obtained for transcriptomic analysis: Baseline (*n* = 5), T1_N (*n* = 3), T1_A (*n* = 2), T1_AW (*n* = 4), T2_N (*n* = 5), T2_A (*n* = 9), T2_AW (*n* = 6), T3_N (*n* = 5), T3_A (*n* = 6), and T3_AW (*n* = 3). Raw RNA-seq reads underwent rigorous quality control using FastQC. After adapter removal using Cutadapt, more than 99% of the total reads met the quality criteria (Phred score ≥ 30), demonstrating high data reliability. Mapping to the Mus musculus reference genome (GRCm38.p6) using STAR resulted in an alignment rate exceeding 98% for most samples. However, sample Base3 exhibited low mapping efficiency and abnormal patterns in junction and gene body coverage, and was therefore excluded from downstream analysis.

### 3.2. Impact of Surgical Wounding on Tumor Progression

**Figure 3:**
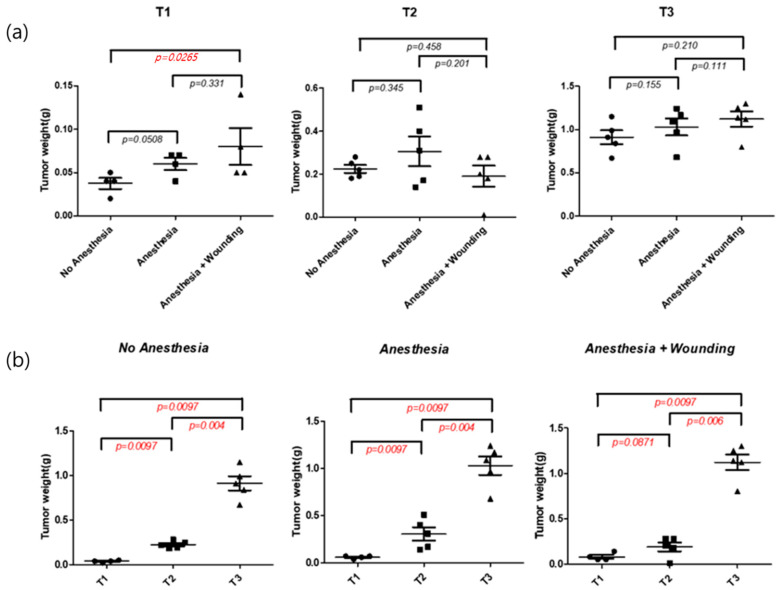
Impact of surgical wounding on ovarian cancer progression in an orthotopic mouse model. Statistical significance between groups is indicated by *p* values shown in the graphs (Student’s *t*-test). Red italic font indicates statistical significance (*p* < 0.05). (**a**) Comparison of tumor weight at postoperative time points T1 (day 1), T2 (week 1), and T3 (week 2) among three experimental groups: no anesthesia, anesthesia alone, and anesthesia plus surgical wounding. (**b**) Longitudinal assessment of tumor growth within each experimental group over time, demonstrating progressive tumor enlargement across all groups as expected.

First, to assess the impact of surgical wounding on ovarian cancer progression, we compared tumor weights across study timepoints (Figure 3). At the early postoperative stage (T1), the Anesthesia plus Wounding (AW) group exhibited a significantly greater tumor burden compared with the No-treatment (N) group (*p* = 0.027). Although statistical significance was not reached at T2 and T3, likely due to increased intragroup variability, the trend toward higher tumor burden in the anesthesia plus wounding group persisted across later time points.

### 3.3. Transcriptomic Alterations Following Wounding at Different Timepoints and Drug Discovery

**Figure 4:**
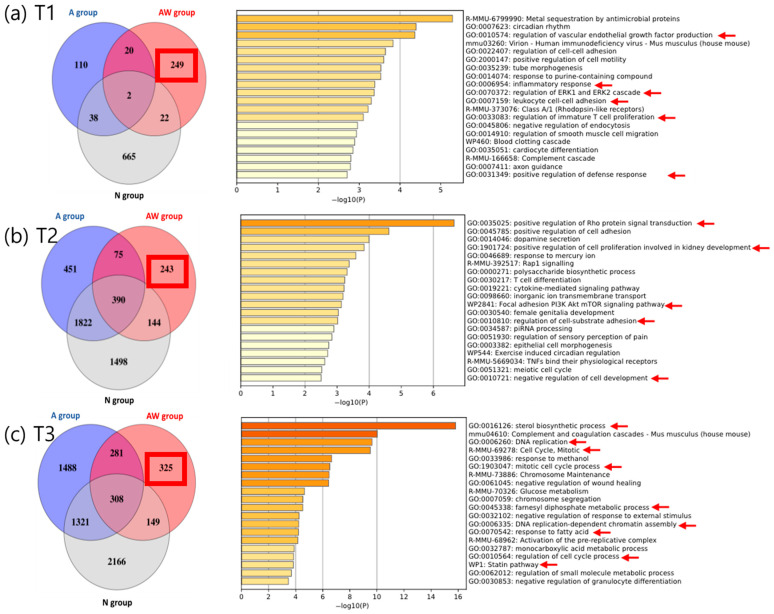
Identification and functional characterization of wound-specific transcriptomic signatures across time points. Venn diagrams illustrate the identification of wound-specific differentially expressed genes (WsDEGs) at T1, T2, and T3. WsDEGs were defined as genes significantly altered in the anesthesia plus wounding (AW) group after explicitly excluding genes overlapping with the no-treatment (N) and anesthesia-only (A) groups, thereby isolating transcriptional changes attributable to surgical wounding rather than anesthesia or baseline tumor progression. Bar graphs depict the top enriched functional pathways derived from these unique WsDEGs based on Metascape analysis. Pathways are ranked by statistical significance (−log10(*p*), and red arrows indicate representative pathways relevant to the dominant biological features observed at each phase. (**a**) At T1, WsDEGs were predominantly enriched in pathways related to acute inflammatory response, immune cell recruitment, and vascular or coagulation-associated processes. (**b**) At T2, enrichment shifted toward pathways associated with cell survival and adaptation, including PI3K–Akt–mTOR signaling and cell adhesion. (**c**) At T3, WsDEGs were mainly enriched in pathways related to cell cycle regulation and lipid metabolic processes, including sterol and cholesterol biosynthesis.

For each timepoint (T1, T2, T3), the distribution of DEGs across the N, A, and AW groups was visualized using Venn diagrams. GO and KEGG pathway enrichment analyses were subsequently performed using wound-specific DEGs uniquely identified in the AW group, and representative enriched pathways are presented in Figure 4. Detailed Venn diagrams and pathway graphs for each experimental group at T1, T2, and T3 are summarized in Supplementary Figures S4–S6.

### 3.4. T1 (One-Day Post-Intervention)

At T1, differential expression analysis comparing AW versus Baseline identified a total of 293 DEGs, comprising 159 up-regulated and 134 down-regulated transcripts. Venn diagram analysis excluding genes overlapping with the N and A groups identified 249 genes as wound-specific transcriptional changes unique to the AW group. The detailed expression patterns and gene lists for these wound-specific signatures are presented in Supplementary Figure S7. Pathway analysis of these 249 WsDEGs revealed significant activation of inflammatory signaling pathways, the blood clotting cascade, VEGF regulation, and leukocyte migration. To explore potential therapeutic strategies to reverse these transcriptomic changes, the REMEDY drug repurposing platform was applied. As a result, Vorinostat (HDAC inhibitor) and Homoharringtonine (protein synthesis inhibitor), which showed negative connectivity scores, were selected as candidates predicted to reverse the wound-induced transcriptional program at T1.

### 3.5. T2 (One-Week Post-Intervention)

At T2, comparison of the AW group with Baseline identified 852 DEGs (341 up-regulated, 511 down-regulated). After removing overlaps with the N and A groups, 243 genes were classified as wound-specific changes at T2. The corresponding heatmaps and gene lists illustrating these stage-specific alterations are provided in Supplementary Figure S8. Pathway analysis of these 243 genes indicated a shift toward growth, survival, and developmental pathways, including PI3K–AKT–mTOR/focal adhesion, Rap1/Rho GTPase signaling, cytokine-mediated signaling, cell–substrate adhesion, epithelial morphogenesis, and T-cell differentiation. In the REMEDY analysis, three drugs emerged as candidates: LY-2090314 (GSK3β inhibitor), Artesunate (DNA-damage/ROS-inducing antimalarial), and Birinapant (SMAC mimetic/XIAP inhibitor). These drugs target survival signaling inhibition, stress/damage response modulation, and apoptosis reactivation, respectively.

### 3.6. T3 (Two-Weeks Post-Intervention)

At T3, a total of 1063 DEGs (349 up-regulated, 714 down-regulated) were identified in the AW group versus Baseline. Excluding genes shared with the N and A groups, 325 genes were confirmed as the specific transcriptional signature. Detailed visualizations of the expression profiles and the full list of these genes are shown in Supplementary Figure S9. These genes were enriched in cell cycle-related programs (DNA replication, mitotic progression, chromosome segregation) and lipid metabolism/biosynthesis (sterol/cholesterol biosynthesis, fatty acid metabolism) pathways. REMEDY analysis identified five candidate drugs to inhibit these processes: Fulvestrant (ER antagonist), AGI-5198 (IDH1 inhibitor), Atorvastatin (HMG-CoA reductase inhibitor), Imatinib (multi-target tyrosine kinase inhibitor), and ABT-737 (BCL-2 family inhibitor). These agents target pathways related to cell cycle regulation, metabolic inhibition, survival signal blockade, and apoptosis induction.

## 4. Discussion

This study utilized a mouse ovarian cancer model to demonstrate that surgical wounding, rather than anesthesia alone, can accelerate tumor growth, particularly in the early phase (T1). We further characterized the accompanying intervention- and stage-specific transcriptomic changes. The inclusion of an anesthesia-only group allowed us to distinguish transcriptional and phenotypic changes attributable to surgical injury from those potentially induced by anesthesia exposure. These transcriptomic analyses suggest that the wound healing response extends beyond simple inflammation to activate cell survival and metabolic pathways, thereby potentially contributing to a transient tumor-promoting microenvironment, particularly during the early postoperative phase that may be less favorable for chemotherapy responsiveness.

The temporal transcriptomic changes observed in this study likely reflect dynamic biological processes within the postoperative tumor microenvironment. Early enrichment of inflammatory and coagulation-related pathways is consistent with the acute wound-healing response, which has been reported to transiently promote tumor cell survival, angiogenesis, and metastatic potential during the perioperative period. As the postoperative period progresses, the shift toward cell adhesion, survival, and metabolic pathways may reflect tumor adaptation to the evolving wound microenvironment. Although our findings are primarily transcriptomic, the observed increase in tumor burden at the early postoperative stage supports the potential phenotypic consequences of these molecular changes.

Clinically, while early initiation of chemotherapy is recommended for patients with residual disease [6,7,20,21,22], previous studies using the ID8 mouse model have reported that administering chemotherapy immediately after surgery (Day 0–1) results in reduced efficacy due to the interference of wound healing signals, whereas administration on Day 7 is most effective [13]. Our findings of hypercoagulation and inflammatory transcriptomic changes at T1 provide molecular context for the observation that the immediate postoperative environment with residual disease may be unfavorable for cytotoxic chemotherapy. These findings provide a biological framework linking perioperative wound responses with clinical observations regarding chemotherapy timing. In particular, they support the concept that the immediate postoperative period may represent a transient biological window during which the tumor microenvironment is less favorable for optimal chemotherapy efficacy.

Acute inflammation is known not only to increase the incidence of ovarian cancer [23,24], but also to be associated with poor prognosis [25]. Accordingly, modulation of inflammatory pathways has been proposed as a strategy to improve oncologic outcomes, and several preclinical and observational studies have suggested potential benefit [26,27,28]. However, clinical trials evaluating conventional anti-inflammatory agents—including NSAIDs, cyclooxygenase-2 inhibitors, and aspirin—have yielded mixed results, underscoring the complexity of inflammation-driven tumor biology and the limitations of non-selective anti-inflammatory approaches [29,30,31].

By integrating RNA-seq analysis with the REMEDY transcriptome-based drug discovery platform, we identified time-dependent wound-induced transcriptional signatures and candidate pathways and compounds predicted to reverse these changes. In the early postoperative phase (T1), Vorinostat emerged as one of promising candidates. Vorinostat is an FDA-approved pan-histone deacetylase inhibitor that has demonstrated anti-inflammatory, anti-angiogenic, and chemosensitizing effects in ovarian cancer models [32,33,34]. Homoharringtonine, another T1-prioritized compound, is a protein translation inhibitor that rapidly depletes short-lived oncogenic proteins, further supports the concept that targeting acute wound-response programs may mitigate surgery-associated tumor-promoting effects [35,36,37]. Together, these findings support the rationale for perioperative drug strategies aimed at transiently modulating early wound-induced biology rather than replacing cytotoxic therapy.

As the postoperative microenvironment progresses into the T2 and T3 phases, transcriptomic programs shift toward stress adaptation, cell survival, proliferation, and metabolic reprogramming. Several candidate agents identified for these later phases target pathways that have been previously investigated in preclinical models as sensitizers or adjuncts to cytotoxic chemotherapy, rather than as standalone therapies. For example, inhibition of stress-response and survival signaling (e.g., GSK3β inhibition [38], or apoptosis modulation [39,40,41]) and targeting of metabolic dependencies [42,43,44,45] have been shown to enhance chemosensitivity or suppress tumor outgrowth in ovarian cancer models [41,46,47,48,49].

This study has several limitations. First, as a preclinical study utilizing a mouse model, it is difficult to definitively conclude that the observed transcriptomic changes are fully recapitulated within the human tumor microenvironment. Some genes identified in the analysis may lack direct human orthologs or exhibit functional interspecies differences, necessitating caution when extrapolating these findings to human disease. In addition, the REMEDY/LINCS framework relies largely on perturbational signatures derived from human cancer cell lines, and cross-species differences between the murine model and human transcriptomic datasets should therefore be considered when interpreting the drug-repurposing results, highlighting the limited direct translational relevance of these findings to human ovarian cancer.

Second, the limited sample size in several subgroups (e.g., T1_A and T3_AW) constrains the statistical interpretation of the data. No genes remained significant after multiple-testing correction using FDR-based methods, and thus the use of raw *p*-values for DEG selection should be interpreted as exploratory with caution.

Third, the experiments were conducted using young mice (6–8 weeks old), whereas epithelial ovarian cancer predominantly occurs in older patients [50]. As aging is known to influence tumor biology, immune responses, and wound healing processes, the transcriptomic responses observed in this model may not fully reflect the aged tumor microenvironment seen in clinical settings.

Furthermore, independent validation of key genes using approaches such as quantitative PCR (qPCR) was not performed in the current study and will be required in future work. Additional functional analyses, such as markers of proliferation, angiogenesis, or immune-cell composition analyses (e.g., deconvolution approaches or flow cytometry), would also strengthen the biological interpretation of these transcriptomic findings. The candidate compounds identified through the REMEDY platform represent transcriptome-based hypotheses and require further experimental validation, including studies in human ovarian cancer cell lines, to confirm their therapeutic relevance.

Nevertheless, this study demonstrates, at the transcriptomic level, that the tumor microenvironment undergoes dynamic temporal changes in the presence of residual disease following surgery, highlighting the immediate postoperative phase as a critical period influencing tumor biology. These results provide foundational data for exploring therapeutic approaches that account for the postoperative biological environment, moving beyond uniform treatment timing and strategies.

## Data Availability

The RNA sequencing data generated in this study is currently being deposited in the Gene Expression Omnibus (GEO) database (Accession number: GSE325548). Processed data supporting the findings of this study are included within the article and its Supplementary Information.

## Supplementary Materials

The following supporting information can be downloaded at: https://www.mdpi.com/article/10.3390/pharmaceutics18040413/s1, Figure S1: Transcriptome-based drug repurposing results for T1 wound-specific DEGs; Figure S2: Transcriptome-based drug repurposing results for T2 wound-specific DEGs; Figure S3: Transcriptome-based drug repurposing results for T3 wound-specific DEGs; Figure S4: Detailed transcriptomic characterization and isolation of wound-specific signatures at the T1 phase; Figure S5: Detailed transcriptomic characterization and isolation of wound-specific signatures at the T2 phase; Figure S6: Detailed transcriptomic characterization and isolation of wound-specific signatures at the T3 phase; Figure S7: Longitudinal expression patterns and top dysregulated genes of the T1 wound-specific signature; Figure S8: Longitudinal expression patterns and top dysregulated genes of the T2 wound-specific signature; Figure S9: Longitudinal expression patterns and top dysregulated genes of the T3 wound-specific signature.

## Abbreviations

The following abbreviations are used in this manuscript:

Abbreviation	Full Term
AAALAC	Association for Assessment and Accreditation of Laboratory Animal Care
A	Anesthesia only
AW	Anesthesia plus Wounding
CORUM	Comprehensive Resource of Mammalian Protein Complexes
DEG	Differentially Expressed Gene
DMEM	Dulbecco’s Modified Eagle’s Medium
EOC	Epithelial Ovarian Cancer
ER	Estrogen Receptor
FC	Fold Change
GO	Gene Ontology
HBSS	Hank’s Balanced Salt Solution
HDAC	Histone Deacetylase
HTSeq	High-Throughput Sequencing
IACUC	Institutional Animal Care and Use Committee
IP	Intraperitoneal
ITS	Insulin-Transferrin-Selenium
KEGG	Kyoto Encyclopedia of Genes and Genomes
KS	Kolmogorov–Smirnov
LINCS	Library of Integrated Network-Based Cellular Signatures
MDS	Multidimensional Scaling
MoA	Mode of Action
N	No Treatment
RNA-seq	RNA Sequencing
RIN	RNA Integrity Number
ROS	Reactive Oxygen Species
SBRI	Samsung Biomedical Research Institute
SMAC	Second Mitochondria-Derived Activator of Caspases
T0	Baseline
Ti	Time of Intervention
T1	Time of 1 Day Post-Intervention
T2	Time of 1 Week Post-Intervention
T3	Time of 2 Weeks Post-Intervention
TPM-UQ	Upper Quartile–Normalized Transcripts Per Million
VEGF	Vascular Endothelial Growth Factor
WsDEG	Wound-Specific Differentially Expressed Gene
