# Supplementary material for: Time-Resolved Transcriptomic Profiling of Surgical Wounds Identifies Stage-Specific Therapeutic Targets for Residual Ovarian Cancer
Source: Pharmaceutics. 2026 Mar 28;18(4):413. doi: 10.3390/pharmaceutics18040413 (PMC13118874; doi:10.3390/pharmaceutics18040413)
Supplement: Supplementary file 1 [file pharmaceutics-18-00413-s001.zip › pharmaceutics-4159500-supplementary.pdf]

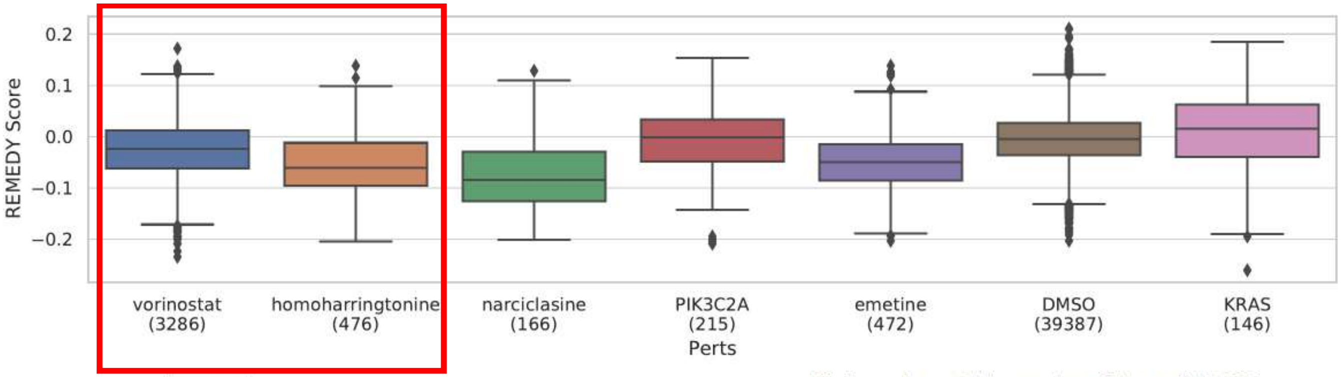

	drug	count	MoA	phase	total	percentage	fisher_pval(log)	TAS_avg
0	vorinostat	13	hdac inhibitor	Launched	3286	0.400	2.330	0.366
1	homoharringtonine	7	protein synthesis inhibitor	Launched	476	1.470	4.690	0.335
2	narciclasine	6	coflilin signaling pathway activator|rho associated kinase activator|lim kinase activator	nan	166	3.610	6.320	0.411
3	PIK3C2A	5	PIK3C2A shRNA	nan	215	2.330	4.430	0.268
4	emetine	5	protein synthesis inhibitor	Phase 2	472	1.060	2.870	0.279
5	DMSO	5	control vehicle	Preclinical	39387	0.010	-0.000	0.093
6	KRAS	4	KRAS cDNA_wt	nan	146	2.740	3.910	0.379

MoA	count
protein synthesis inhibitor	2
hdac inhibitor	1
coflilin signaling pathway activator|rho associated kinase activator|lim kinase activator	1
PIK3C2A shRNA	1
control vehicle	1
KRAS cDNA_wt	1

Direction	Counts
Up	66 over 143
Down	54 over 108

Figure S1. Transcriptome-based drug repurposing results for T1 wound-specific DEGs

Candidate compounds predicted to reverse the wound-specific transcriptional signature at T1 (1 day post-surgery) were identified using the REMEDY platform. The boxplot shows the distribution of REMEDY connectivity scores for representative perturbagens, where negative scores indicate a predicted reversal of the wound-induced gene expression pattern. Vorinostat and homoharringtonine were prioritized as candidate compounds based on their enrichment within the top-ranked perturbagen signatures. The table summarizes the ranking results, including the number of occurrences within the top signatures, mechanism of action (MoA), clinical phase, and enrichment statistics. MoA enrichment analysis highlighted protein synthesis inhibition and HDAC inhibition as the dominant therapeutic mechanisms predicted to counteract the T1 wound-associated transcriptomic program. The direction counts indicate the number of up- and down-regulated genes included in the query signature.

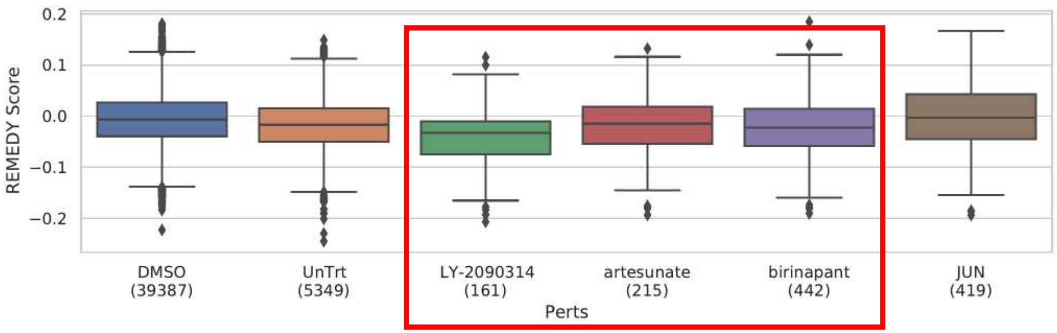

	drug	count	MoA	phase	total	percentage	fisher_pval(log)	TAS_avg
0	DMSO	9	control vehicle	Preclinical	39387	0.020	-0.000	0.093
1	UnTrt	6	UnTrt ctl_untrt	nan	5349	0.110	0.050	0.229
2	LY-2090314	5	glycogen synthase kinase inhibitor	Phase 2	161	3.110	5.030	0.402
3	artesunate	4	dna synthesis inhibitor	Launched	215	1.860	3.280	0.175
4	birinapant	4	xiap inhibitor	Phase 2	442	0.900	2.150	0.122
5	JUN	4	JUN shRNA	nan	419	0.950	2.230	0.281

MoA	count
control vehicle	1
UnTrt ctl_untrt	1
glycogen synthase kinase inhibitor	1
dna synthesis inhibitor	1
xiap inhibitor	1
JUN shRNA	1

Direction	Counts
Up	84 over 134
Down	47 over 109

Figure S2. Transcriptome-based drug repurposing results for T2 wound-specific DEGs

Using the same REMEDY-based analysis described in Figure X, candidate compounds predicted to reverse the T2 (1 week post-surgery) wound-specific transcriptional signature were identified. LY-2090314, artesunate, and birinapant were prioritized among the top-ranked perturbagen signatures. The accompanying tables summarize the ranking statistics, mechanisms of action (MoA), and DEG direction counts used in the query signature.

	drug	count	MoA	phase	total	percentage	fisher_pval(log)	TAS_avg
0	torin-1	16	pi3k inhibitor|mtor inhibitor	Preclinical	694	2.310	12.800	0.204
1	MK-2206	9	akt inhibitor	Phase 2	1117	0.810	3.830	0.129
2	THZ-2-98-01	8	nan	nan	198	4.040	8.610	0.180
3	GDC-0941	7	pi3k inhibitor	Phase 2	1045	0.670	2.650	0.152
4	WYE-125132	6	mtor inhibitor	Preclinical	585	1.030	3.260	0.145
5	fulvestrant	6	estrogen receptor antagonist	Launched	867	0.690	2.410	0.132
6	AGI-5198	5	isocitrate dehydrogenase inhibitor	Preclinical	363	1.380	3.370	0.034
7	MELK	4	MELK shRNA	nan	228	1.750	3.180	0.233
8	EGFR	4	EGFR shRNA	nan	328	1.220	2.600	0.251
9	CEBPB	4	CEBPB shRNA	nan	30	13.330	6.670	0.243
10	ATG16L1	4	ATG16L1 shRNA	nan	50	8.000	5.760	0.220
11	atorvastatin	4	hmgcr inhibitor	Launched	527	0.760	1.890	0.112
12	imatinib	4	kit inhibitor|abl kinase inhibitor|pdgfr inhibitor|bcr-abl kinase inhibitor|pdgfr tyrosine kinase receptor inhibitor	Launched	843	0.470	1.250	0.103
13	ABT-737	4	bcl inhibitor	hase 1/Phase 2	774	0.520	1.360	0.121

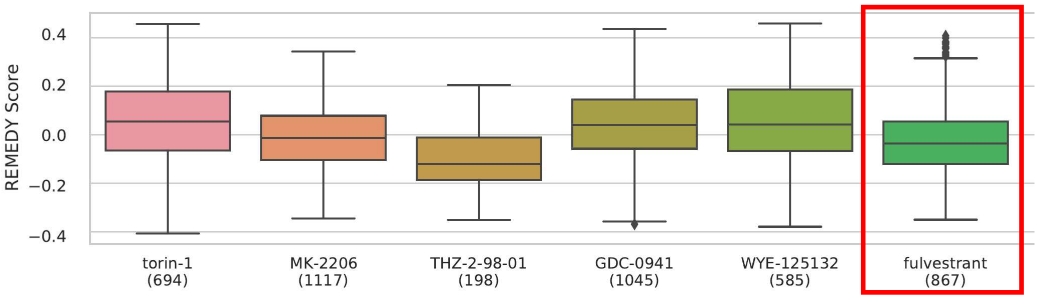

MoA	count
pi3k inhibitor|mtor inhibitor	1
akt inhibitor	1
pi3k inhibitor	1
mtor inhibitor	1
estrogen receptor antagonist	1
isocitrate dehydrogenase inhibitor	1
MELK shRNA	1
EGFR shRNA	1
CEBPB shRNA	1
ATG16L1 shRNA	1
hmgcr inhibitor	1
kit inhibitor|abl kinase inhibitor|pdgfr inhibitor|bcr-abl kinase inhibitor|pdgfr tyrosine kinase receptor inhibitor	1
bcl inhibitor	1

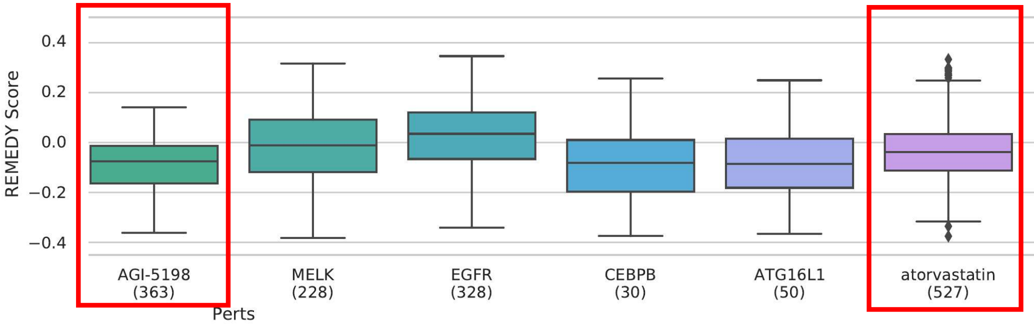

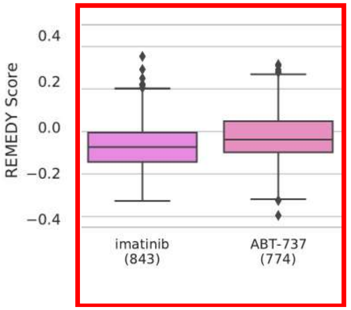

Direction	Counts
Up	70 over 131
Down	121 over 192

Figure S3. Transcriptome-based drug repurposing results for T3 wound-specific DEGs
Using the same REMEDY-based analysis described in the previous figures, candidate compounds predicted to reverse the T3 (2 weeks post-surgery) wound-specific transcriptional signature were identified. Fulvestrant, AGI-5198, atorvastatin, imatinib, and ABT-737 were prioritized among the top-ranked perturbagen signatures. The accompanying tables summarize the ranking statistics and mechanisms of action (MoA), highlighting estrogen receptor antagonism, isocitrate dehydrogenase inhibition, HMGCR inhibition, and kinase inhibition as potential mechanisms associated with reversal of the T3 wound-associated transcriptomic program. Direction counts indicate the number of up- and down-regulated genes included in the query signature.

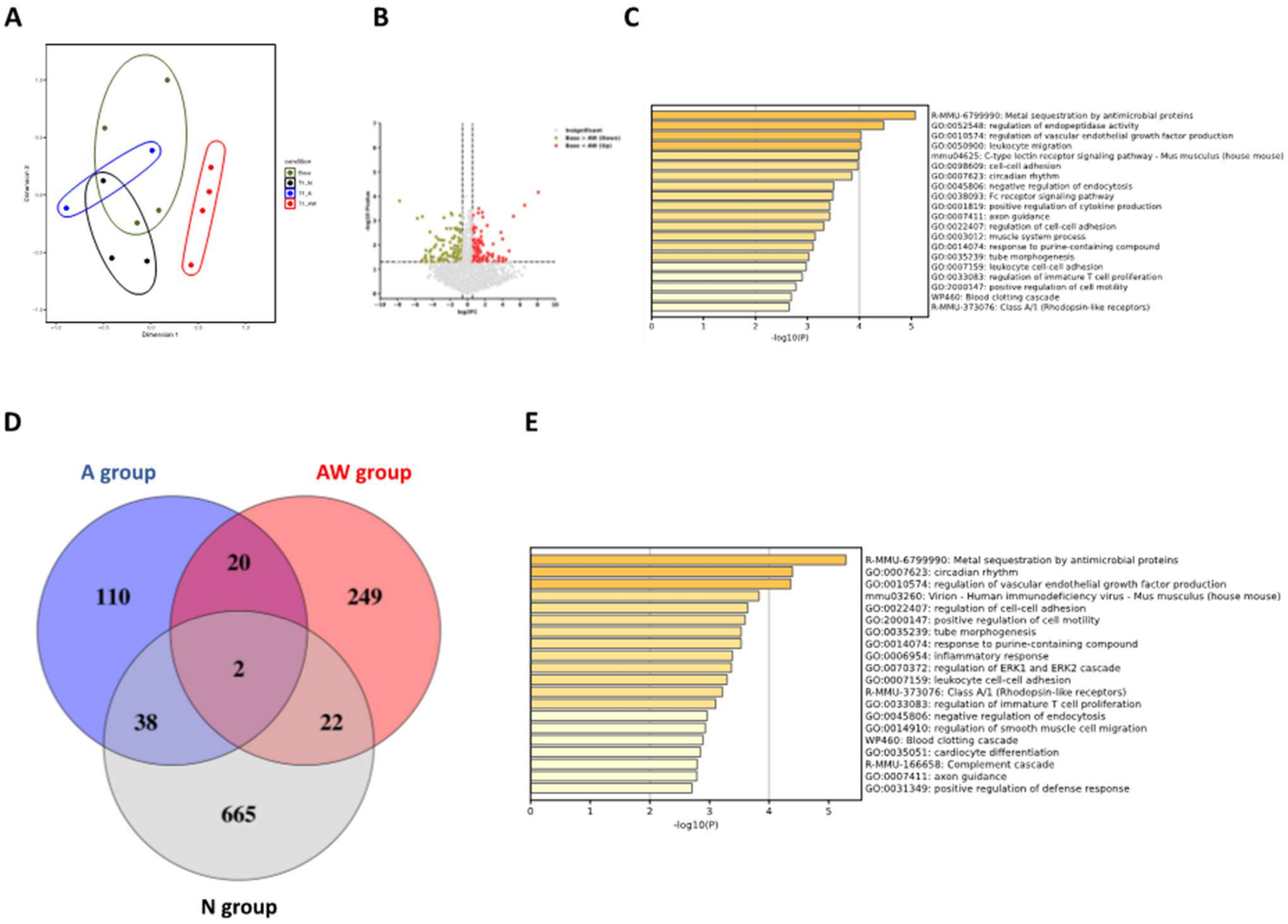

Figure S4. Detailed transcriptomic characterization and isolation of wound-specific signatures at the T1 phase

(a) Multidimensional Scaling (MDS) plot visualizing transcriptomic variations among groups at T1. The Anesthesia + Wounding (AW, red) group formed a distinct cluster separated from the Baseline (Base), No-treatment (N), and Anesthesia (A) groups, indicating a unique transcriptomic shift induced immediately by surgical wounding.

(b) Volcano plot displaying differentially expressed genes (DEGs) in the AW group versus Baseline. A total of 293 DEGs (159 up-regulated, 134 down-regulated) were identified ($|Log2FC| \geq 0.58$, $p \leq 0.05$).

(c) Functional enrichment analysis of total DEGs in the AW group (before filtering). Top enriched terms included inflammatory response, regulation of vascular endothelial growth factor (VEGF) production, and blood clotting cascade, reflecting the acute reaction to tissue injury.

(d) Venn diagram isolating wound-specific DEGs (WsDEGs). By explicitly excluding genes overlapping with the N and A groups, 249 genes were identified as signatures unique to surgical wounding.

(e) Bar graph of functional pathways enriched in the 249 WsDEGs. The analysis confirms the dominance of acute inflammatory and coagulation-related pathways as the primary phenotype of the T1 phase.

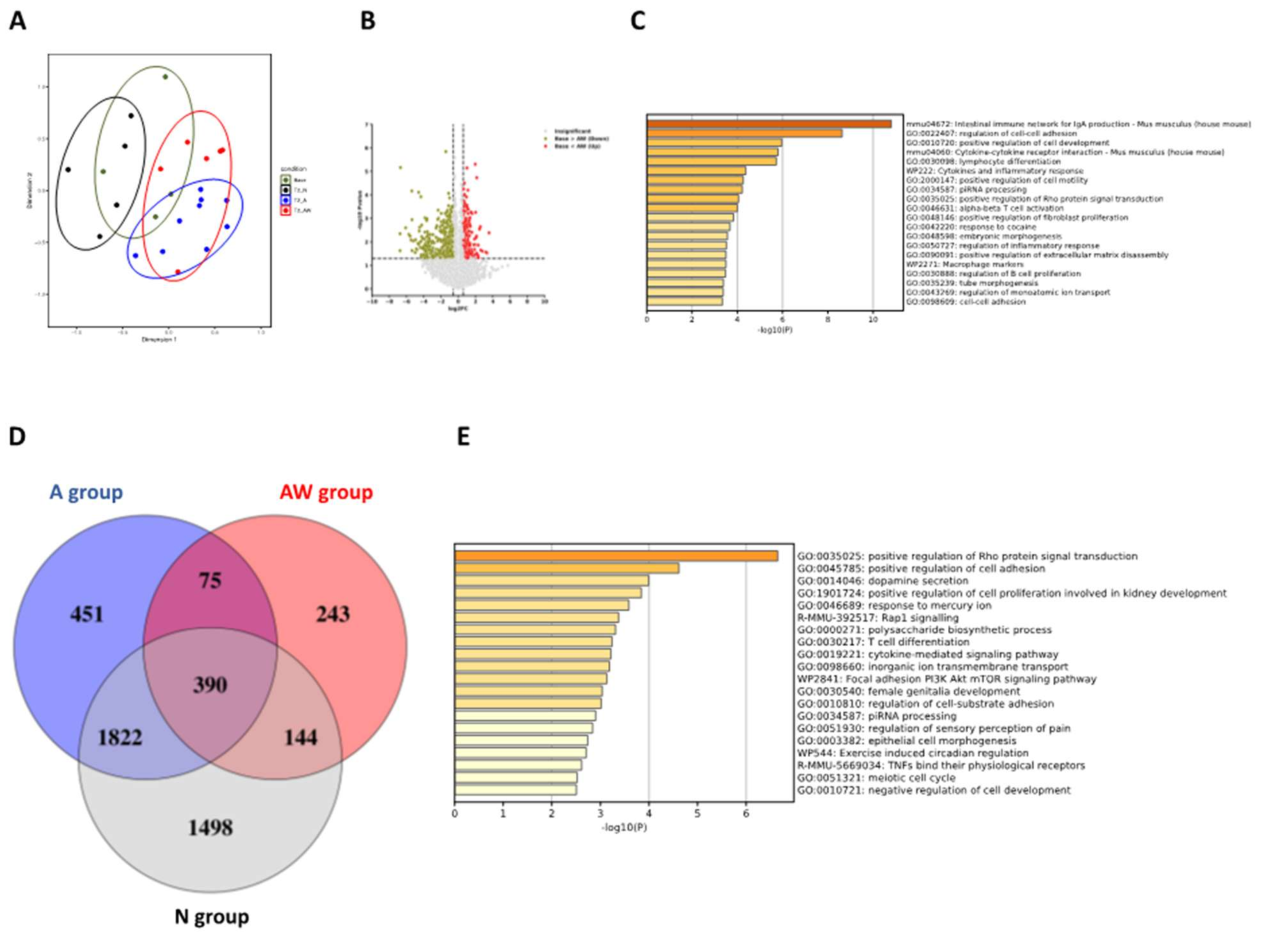

Figure S5. Detailed transcriptomic characterization and isolation of wound-specific signatures at the T2 phase

(a) Multidimensional Scaling (MDS) plot visualizing transcriptomic similarities among groups at T2. The Baseline (Base), No-treatment (N), Anesthesia (A), and Anesthesia + Wounding (AW) groups showed partial clustering, particularly between A and AW, indicating shared transcriptomic features at this intermediate phase.

(b) Volcano plot of differentially expressed genes (DEGs) in the AW group versus Baseline. A total of 852 DEGs (341 up-regulated, 511 down-regulated) were identified (|Log2FC| ≥ 0.58, p ≤ 0.05).

(c) Functional enrichment analysis of total DEGs in the AW group (before filtering). Major enriched pathways included inflammatory and immunological responses (e.g., cytokine-cytokine receptor interaction, lymphocyte differentiation) and extracellular matrix remodeling.

(d) Venn diagram isolating wound-specific DEGs (WsDEGs). By excluding genes overlapping with the N and A groups, 243 genes were identified as signatures unique to surgical wounding.

(e) Bar graph of functional pathways enriched in the 243 WsDEGs. The analysis highlights a shift toward cell growth, survival, and signaling, including Rap1 signaling and the Focal adhesion-PI3K-Akt-mTOR signaling pathway.

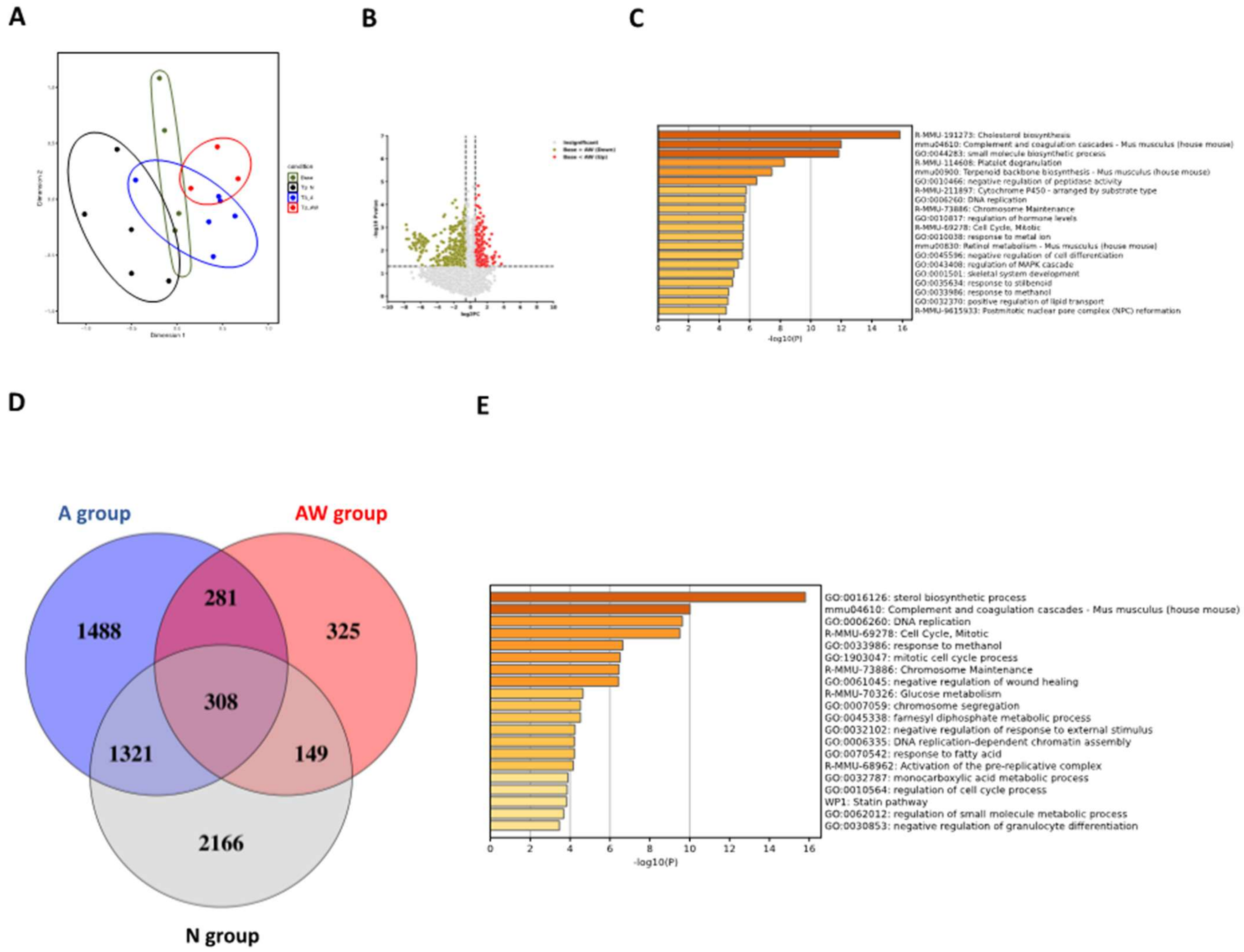

Figure S6. Detailed transcriptomic characterization and isolation of wound-specific signatures at the T3 phase

(a) Multidimensional Scaling (MDS) plot at T3. Groups showed partial clustering, with some overlap between Anesthesia (A) and No-treatment (N) groups.
(b) Volcano plot displaying DEGs in the AW group versus Baseline. A distinct increase in transcriptional alteration was observed, with 1,063 DEGs (349 up-regulated, 714 down-regulated) identified.
(c) Functional enrichment analysis of total DEGs. Enriched terms were dominated by lipid metabolism (cholesterol biosynthesis) and cell cycle processes (DNA replication, chromosome maintenance).
(d) Venn diagram identifying 325 wound-specific DEGs (WsDEGs) after excluding overlaps with N and A groups.
(e) Bar graph of functional pathways enriched in the 325 WsDEGs. The signature confirms a metabolic and proliferative phenotype, with significant enrichment of sterol/cholesterol biosynthetic processes, DNA replication, and mitotic cell cycle regulation

A

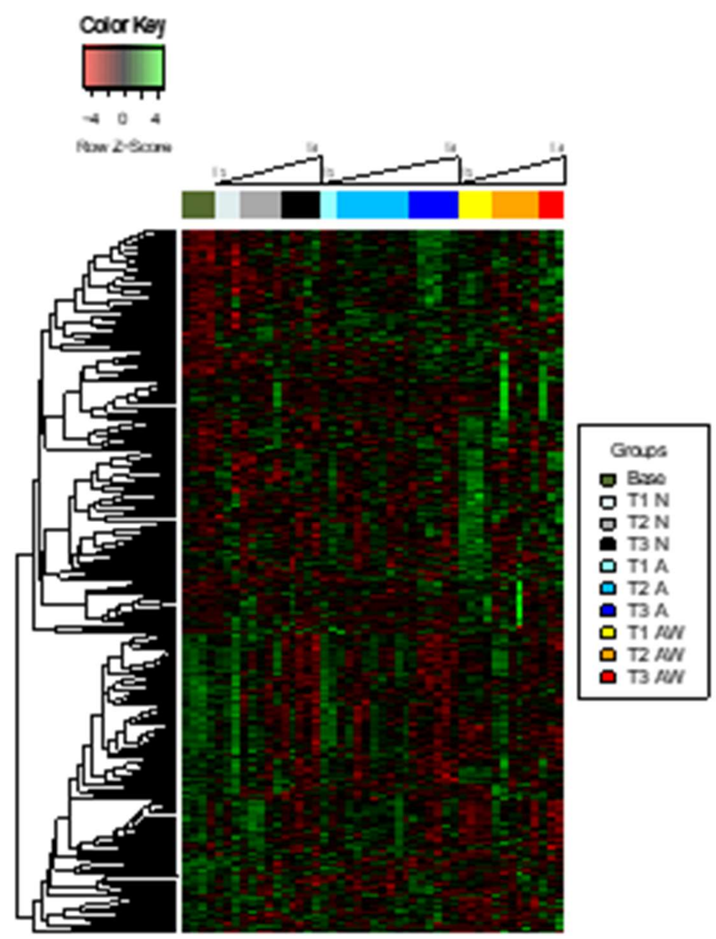

B

Symbol	Description	log2FC
F9	coagulation factor IX	4.76
Gm4788	predicted gene 4788	4.41
Isx	intestine specific homeobox	4.05
Hoxd11	homeobox D11	4.02
Fhl5	four and a half LIM domains 5	4.02
Gm14391	predicted gene 14391	-4.07
Mab21l4	mab-21-like 4	-4.12
Rnase13	ribonuclease, RNase A family, 13 (non-active)	-4.16
Cdh17	cadherin 17	-4.18
Tgm7	transglutaminase 7	-4.20
Alox15	arachidonate 15-lipoxygenase	-4.33
Prok2	prokineticin 2	-4.72
Gm614	predicted gene 614	-4.73
Vmn2r26	vomeronasal 2, receptor 26	-4.74
Gbp2b	guanylate binding protein 2b	-4.80
Cd209b	CD209b antigen	-4.86
Stfa1	stefin A1	-5.17

Figure S7. Longitudinal expression patterns and top dysregulated genes of the T1 wound-specific signature.

(a) Heatmap illustrating the expression levels of T1 WsDEGs across all time points (T1, T2, T3) and groups. Hierarchical clustering revealed three major patterns: a cluster increasing over time, a cluster with no specific pattern, and a large cluster showing decreasing expression over time.

(b) List of top differentially expressed genes within the T1 wound-specific signature. F9 (Coagulation Factor IX) was identified as the most significantly up-regulated gene (Log2FC = 4.76), consistent with the hypercoagulation phenotype, while Stfa1 was the most down-regulated gene.

A

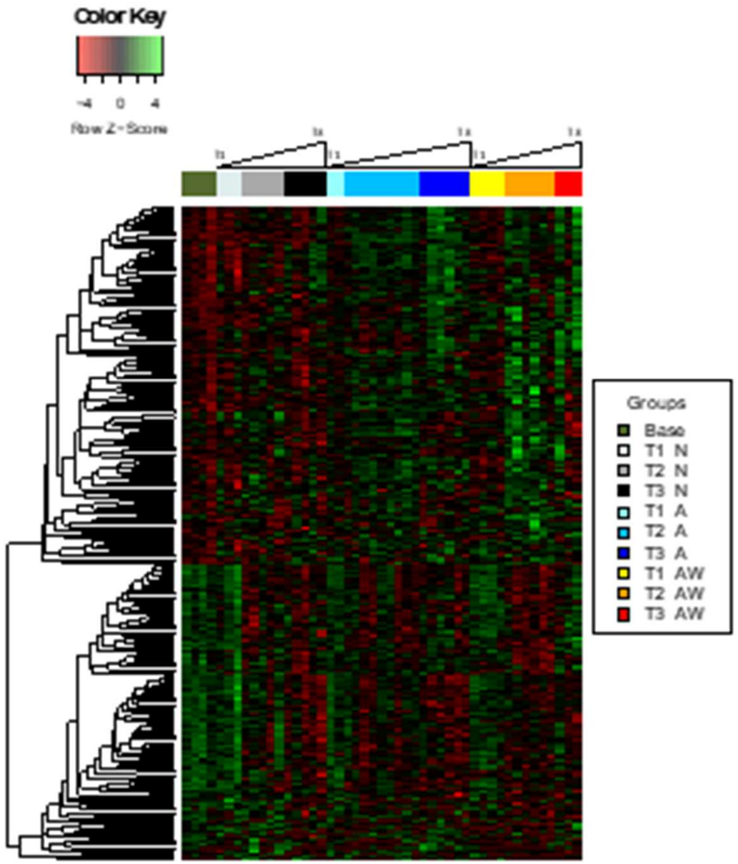

B

Symbol	Description	log2FC
Sfta2	surfactant associated 2	-4.05
Rln3	relaxin 3	-4.06
Fkbp6	FK506 binding protein 6	-4.10
Arl14	ADP-ribosylation factor-like 14	-4.11
Otos	otospiralin	-4.21
H2ac4	H2A clustered histone 4	-4.30
Gm10563	predicted gene 10563	-4.31
Pmis2	PMIS2 transmembrane protein	-4.38
Tssk3	testis-specific serine kinase 3	-4.58
Pla2g2f	phospholipase A2, group IIF	-4.67
Scgb1a1	secretoglobin, family 1A, member 1 (uteroglobin)	-4.76
Obp2a	odorant binding protein 2A	-4.86
Ccl1	chemokine (C-C motif) ligand 1	-4.98
Gm17606	predicted gene, 17606	-5.31
Scgb2b7	secretoglobin, family 2B, member 7	-5.36
Gm10800	predicted gene 10800	-6.74

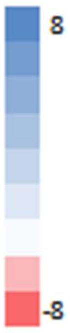

Figure S8. Longitudinal expression patterns and top dysregulated genes of the T2 wound-specific signature.

(a) Heatmap showing the temporal expression of T2 WsDEGs. Clustering identified two major patterns: a cluster showing increasing expression over time (especially in A and AW groups) and a cluster showing decreasing pattern.

(b) List of top differentially expressed genes within the T2 wound-specific signature. Notable down-regulated genes included Sfta2, Rln3, and Scgb2b7 (Log2FC = -5.36)

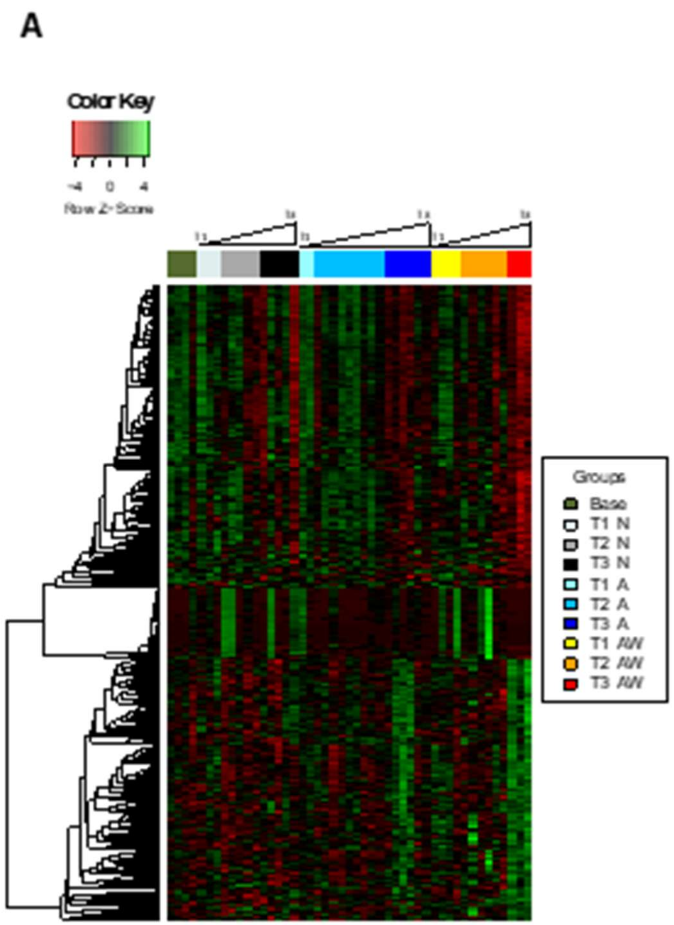

B

Symbol	Description	log2FC
Rnf17	ring finger protein 17	-4.02
Tnni3k	TNNI3 interacting kinase	-4.03
F5	coagulation factor V	-4.06
Gm42878	predicted gene 42878	-4.09
Papolb	poly (A) polymerase beta	-4.12
Gucy2c	guanylate cyclase 2c	-4.17
Ffar1	free fatty acid receptor 1	-4.36
Ttc6	tetratricopeptide repeat domain 6	-4.44
Prg4	proteoglycan 4	-4.46
Myo3b	myosin IIIB	-4.53
Skint3	selection and upkeep of intraepithelial T cells 3	-4.77
Lep	leptin	-5.33
Cyp3a11	cytochrome P450, family 3, subfamily a, polypeptide 11	-5.79
Pon1	paraoxonase 1	-5.84
Serpina1d	serine (or cysteine) peptidase inhibitor, clade A, member 1D	-5.86
Serpina1e	serine (or cysteine) peptidase inhibitor, clade A, member 1E	-5.99
Serpina1c	serine (or cysteine) peptidase inhibitor, clade A, member 1C	-6.04
Serpina1b	serine (or cysteine) preptidase inhibitor, clade A, member 1B	-6.12
Fabp1	fatty acid binding protein 1, liver	-6.30

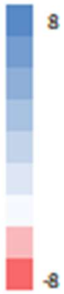

Figure S9. Longitudinal expression patterns and top dysregulated genes of the T3 wound-specific signature.

(a) Heatmap displaying the expression dynamics of T3 WsDEGs. Three clusters were observed, including patterns of increasing expression across time in the Anesthesia (A) and AW groups.

(b) List of top differentially expressed genes within the T3 wound-specific signature. Fabp1 (Fatty acid binding protein 1) was identified as the most significantly down-regulated gene (Log2FC = -6.30), potentially reflecting the altered lipid metabolic state.
